# Differential Foreign Body Reactions between Branched and Linear Glucomannan Scaffolds

**DOI:** 10.3390/jfb13040293

**Published:** 2022-12-11

**Authors:** Yuwei Li, Yu Liu, Senio Campos de Souza, Tzuwei Chao, Lei Dong, Guoxing Sun, Chunming Wang, Yiming Niu

**Affiliations:** 1 State Key Laboratory of Quality Research in Chinese Medicine, Institute of Chinese Medical Sciences, Department of Pharmaceutical Sciences, Faculty of Health Science, University of Macau, Taipa, Macau SAR 999078, China; 2 Faculty of Health Sciences, University of Macau, Macau SAR 999078, China; 3 State Key Laboratory of Pharmaceutical Biotechnology, School of Life Sciences, Nanjing University, Nanjing 210093, China; 4 Joint Key Laboratory of the Ministry of Education, Institute of Applied Physics and Materials Engineering, University of Macau, Avenida da Universidade, Taipa, Macau SAR 999078, China; 5 Zhuhai UM Science & Technology Research Institute (ZUMRI), Hengqin, Zhuhai 519031, China

**Keywords:** glucomannan, polysaccharide structure, electrospun scaffolds, immune response, foreign body reaction

## Abstract

The extent and patterns of foreign body reaction (FBR) influence the function and feasibility of biomaterials. Polysaccharides, as an important biomaterial category, have received increasing attention in diverse biomaterials design and biomedical applications due to their excellent polymeric and biocompatible characteristics. Their biological effects are usually associated with their monosaccharide composition or functional groups, yet the contribution of their glycan structure is still unknown. Herein, two glucomannans, similar in composition and molecular weight with differences in glycan structure, linear-chain (Konjac glucomannan, KGM), and branched-chain (*Bletilla striata* polysaccharide, BSP), were adopted to explore the host–biomaterials interaction. After acetyl modification, these polysaccharides were fabricated into electrospun scaffolds to reduce the impacts derived from the physical properties and surface morphology. According to a systematic study of their biological effects on immune cells and host response in a subcutaneous implantation model in vivo, it was revealed that acetyl KGM (acKGM) scaffolds caused a stronger FBR than acetyl BSP materials. Additionally, acKGM could stimulate macrophages to release pro-inflammatory cytokines, suggesting the influence of sugar chain arrangement on FBR and providing clues for the fine regulation of immune response and novel biomaterials design.

## 1. Introduction

Recent progress in biomaterials sciences exploring medical implants, tissue scaffolds, and drug carriers has highlighted the importance of tissue–materials interactions, which can significantly influence or even determine the outcome and fate of each application [1,2,3]. Once implanted in vivo, biomaterials elicit a series of responses involving multiple immune cell types and other biological components, widely referred to as foreign body reactions (FBR) [4,5,6]. The extent and patterns of FBR are determined by chemical (e.g., substance type, hydrophobicity, surface charge, etc.) and physical (e.g., substrate stiffness, surface topography, etc.) properties of the materials [7]. These factors directly influence the feasibility of using novel biomaterials in clinical practice. As such, understanding how our immune system triggers FBRs following implantation has become critically important in the development of new functional biomaterials.

In recent years, polysaccharides have gained considerable attention in the developing of novel biomaterials [8,9,10]. These polysaccharides have been shown to possess a variety of biological activities, including antioxidant [11,12], anti-tumor [13,14], and immunoregulatory [15,16] functions. Many of these functions have been correlated with its chemical composition, such as the presence of specific sugar units that are easily recognized by cell surface receptors [17].

Among the different types of polysaccharides, there has been great interest in exploring glucomannans (GM) for the development of functional biomaterials due to their high stability, water retention and favorable gel properties [18,19,20]. We decided to isolate the linear glucomannan based on Konjac glucomannan (KGM) and the branched glucomannan based on *Bletilla striata* polysaccharide (BSP) since they can both be extracted from natural sources. Having identified the structure and structure–activity relationships of these materials, they have been developed as orthopedic and vascular biomaterials. In our previous study, acetyl-modified BSP showed distinctive monocyte/macrophage affinity to support macrophage adhesion, and more importantly, it efficiently induced the macrophage activation to express pro-osteogenic/-angiogenic cytokines [21]. In another follow-up study, a mono compositional scaffold based on BSP with anisotropic porosity was designed to direct an endogenous bone repair in situ mediated by the paracrine power of mobilized macrophages, which proposed a possible solution for comprehensive biological functions based on a simple design with minimal demands for exogenous components [22]. Mu et al. designed a “Bridge-Building” glycan scaffold based on KGM mimicking microbial invasion for in situ endothelialization, representing an effective, safe, and alternative strategy for ischemic vascular therapy [23]. More recently, attempts based on the 3D printing technique have been made to solve the processing problem of polysaccharides and boost the therapeutic applications of these materials. For instance, a nonsolvent quenching strategy was developed to overcome the poor shape fidelity of these polysaccharide materials during 3D printing without extra chemical modification or blending with other ingredients [24]. However, it remains largely unknown how the structural differences of BSP and KGM, a branched and a linear polysaccharide, will trigger different FBRs and bring about a different biological activity.

This study explores the importance of structural arrangement in triggering immune responses. Two natural polysaccharides, linear-chain KGM and branched-chain BSP, were selected due to their similarities in composition and molecular weight [25,26]. Modification by acetylation was performed to increase their biological activity and hydrophobicity to facilitate scaffold preparation. The 3D scaffolds were produced via electrospinning that enables a controllable surface morphology using KGM and BSP with a comparable degree of acetylation. A systematic study was performed using these two scaffolds to explore their effects on immune and non-immune cells in vitro and how these trigger FBR in vivo. Surprisingly, our results showed that acetyl KGM (acGM) caused a stronger FBR than acetyl BSP (acBSP) in vivo. Besides, acBSP was better absorbed in the body when compared to acGM. Our results suggest that the structural differences in sugar chain arrangement impact the triggered immune response.

## 2. Materials and Methods

### 2.1. Materials

Konjac glucomannan (KGM) was provided by Shimizu Chemical Corporation (Mihara, Japan). The dry roots of *Bletilla striata* were acquired in Guizhou, China. ELISA kit for mouse IL-1β was purchased from Neobioscience (Shenzhen, China). ELISA kit for mouse TNF-α was purchased from Invitrogen (Waltham, MA, USA). The primary antibodies, rabbit anti-CD31 and rabbit anti-TNF-α, were purchased from Abcam (Cambridge, UK). The secondary antibody Alexa Fluor 488-conjugated anti-rabbit was purchased from Cell Signaling Technology (Danvers, MA, USA). TFAA and other chemicals were purchased from Sigma-Aldrich (St. Louis, MO, USA).

### 2.2. Synthesis of Acetyl Glucomannan

*Bletilla striata* polysaccharide (BSP) was derived from roots from *Bletilla striata* using a previously reported protocol [27]. BSP was extracted with water. After repeated deproteinization using Sevag’s solution, the polysaccharide was precipitated in ethanol, dissolved in water, and dialyzed (MWCO, 10 kDa) for three days. The product was lyophilized to obtain BSP. Acetyl modification to obtain acBSP was performed using another previously reported protocol [23]. The pulverized BSP powder (400 mg) was uniformly dispersed in 40 mL of pyridine and stirred at 90 °C for 30 min. Then, a 5 mL mixture of acetic anhydride and pyridine (1:1, *v*/*v*) was added dropwise to the reaction vessel. After 12 h, the reaction was stopped with the addition of milli-Q water (20 mL). The samples were then precipitated with 4× volume of ethanol overnight. The product was then dissolved in chloroform, washed three times with ethanol, and dried in vacuo to obtain acBSP.

KGM was dialysed against the membrane with molecular weight cut-off (MWCO) of 10 kDa and lyophilised to remove small molecule impurities and inorganic salts. Acetylation of KGM was carried out according to a reported protocol [28]. A premixed solution of 10 mL of trifluoroacetic anhydride (TFAA) and 5 mL of acetic acid (2:1, *v*/*v*) was stirred at 50 °C for 20 min. Then 300 mg of the pulverized KGM powder was added to the reaction system and stirred at 50 °C for 3.5 h. The product was then treated in the same way used to obtain acBSP (i.e., the product was precipitated with ethanol, dissolved in chloroform, washed with ethanol, and dried in vacuo to obtain acGM.)

### 2.3. Characterization of acGM and acBSP

The acGM and acBSP were characterized by FTIR spectrum and ^1^H-NMR (600 MHz). The acGM, acBSP, and BSP were dissolved in dimethyl sulfoxide (DMSO) (δ 2.50 ppm), while GM was dissolved in D_2_O (δ 4.80 ppm).

The degree of acetylation was determined by hydroxylamine-ferric trichloride method. In short, a serial dilution of β-D-acylated glucose solution was used to obtain a standard curve, and the degree of substitution (DS) was calculated as follow: DS = 162 × N/(1 – 42 × N), where N represents the amount-of-substance of acetyl group per unit mass of the product (mol/g) [23].

### 2.4. Preparation of Electrospun Scaffolds

For electrospinning, 10% solutions of acGM or acBSP were prepared in a spinning co-solvent (chloroform: DMF = 2:1, *v*/*v*). Electrospinning was then carried out on an ES instrument (TL-Pro, Tongliweina Co., Shenzhen, China) with the following parameters: negative voltage: −4 kV; positive voltage: +12 kV; humidity: <30%; temperature: 37 °C; rate: 0.5 mL/h; total volume: 0.75 mL.

### 2.5. Contact Angle Measurement

The testing materials were prepared following the abovementioned protocol. Contact angles were measured by the contact angle instrument (JC-2000D1, Powereach Co., Shanghai, China).

### 2.6. Scanning Electron Microscopy (SEM) and Quantification of Electrospun Scaffolds

The acGM and acBSP ES scaffolds structures were assessed by scanning electron microscopy (JSM–6700F, JEOL, Akishima, Japan). Following gold sputter coating, images of electrospinning fiber were captured.

Pore size, fiber diameter, and porosity were quantified from SEM images of acGM and acBSP ES scaffolds. The pore size and fiber diameter within an image for one sample (n = 3) were quantified using ImageJ.

Images were converted to binary format using ImageJ and the threshold was adjusted, where the fibers appeared red and the pores black. The percentage of the black area relative to each image’s total scaffold surface area was defined as the percent porosity.

### 2.7. Cell Culture

The murine macrophage cell line RAW264.7 was purchased from Stem Cell Bank, Chinese Academy of Sciences. The mouse fibroblast cell line NIH/3T3 was purchased from Procell Life Science & Technology Co., Wuhan, China. Following the supplier’s instructions, RAW264.7 and NIH/3T3 were cultured in DMEM medium supplemented with 10% fetal bovine serum (FBS) and 1% penicillin-streptomycin at 37 °C with 5% CO_2_.

### 2.8. Cell Adhesion and Proliferation on the Electrospun Scaffolds

RAW264.7 cells (1.5 × 10^4^ cells per well) and NIH-3T3 cells (1.5 × 10^4^ cells per well) were seeded onto electrospun scaffolds in a 24-well plate and incubated for three days. The scaffolds were stained with Live/Dead staining dyes (Live, Calcein AM; Dead, PI) and F-actin dyes as per the manufacturer’s instructions. Cell proliferation was tested using the EdU Cell Proliferation Kit as per the manufacturer’s instructions (the monitoring time was 12 h). Fluorescence microscopy (Dmi8, Leica) was performed to observe cell morphology.

CCK-8 proliferation assay was performed to produce the cells’ survival curve. RAW264.7 (5 × 10^3^ cells/well) and NIH-3T3 (5 × 10^3^ cells/well) were seeded onto electrospun scaffolds. On days 1, 3, 5, and 7, the CCK8 reagent was added to each well and incubated at 37 °C for 3–4 h. The absorbance was measured at 450 nm using a microplate reader (SpectraMax M5) in accordance with the manufacturer’s instructions.

### 2.9. Enzyme-Linked Immunosorbent Assay (ELISA)

The culture medium of RAW264.7 and NIH/3T3 were collected to quantify the expression levels of TNF-α and IL-1β using ELISA assay kits as per the manufacturers’ instructions.

### 2.10. Real-Time Quantitative PCR

Real-Time Quantitative PCR (RT-qPCR) was performed with Mx3005P qPCR System (Agilent, Santa Clara, CA, USA). All primers used were synthesized by Life Technologies (Shanghai, China): mNos2 (F) CCAAGCCCTCACCTACTTCC, mNos2 (R) CTCTGAGGGCTGACACAAGG, mArg-1 (F) CAGAAGAATGGAAGAGTCAG, mArg-1 (R) CAGATATGCAGGGAGTCACC, mCOL1 (F) CAACAGTCGCTTCAC CTACAGC, mCOL1 (R) GTGGAGGGAGTTTACACGAAGC, m-αSMA (F) GG CACCACTGAACCCTAAGG, m- αSMA (R) ACAATACCAGTTGTACGTCCAGA, m-βactin (F) GCTGGTCGTCGACAACGGCTC, m-βactin (R) CAAACATGATC TGGGTCATCTTTTC, mGAPDH (F) AACGACCCCTTCATTGAC, mGAPDH (R) TCCACGACATACTCAGCAC. RAW264.7 cells (1.2 × 10^6^ cells per well) and NIH-3T3 cells (1.2 × 10^6^ cells per well) were seeded onto electrospun scaffolds in a 6-well plate and incubated. After three days of incubation, the total RNA of each cell type (2 wells per cell type) was extracted with Trizol reagent. The cDNA synthesis was then performed using a reverse transcription kit (Promega, Madison, WI, USA) as per the manufacturer’s instructions.

### 2.11. In Vivo Subcutaneous Implantation and Histological Evaluation

The animal experiment was conducted according to the protocol approved by the Animal Ethics Committee, University of Macau (UMARE-006-2022). The electrospun scaffolds were trimmed into 1 cm × 0.2 cm rectangle. A small incision was made symmetrically on either side of the back of each mouse, the biomaterial was inserted after blunt dissection, and the wound sites were sutured. On days 7 and 14, three mice were sacrificed. On each mouse, the material with its overlying skin was excised. The collected tissues were fixed with 4% paraformaldehyde overnight, embedded in paraffin, sectioned, and stained with hematoxylin-eosin (HE) staining kit according to the manufacturer’s instructions.

### 2.12. Immunofluorescence Staining

The tissue sections were deparaffinized, rehydrated, operated with antigen retrieval, blocked with BSA (5%, 45 min), and incubated with primary antibodies at 4 °C overnight. The primary antibodies used include anti-CD31 and anti-TNF-α (Abcam). Next, the sections were incubated with secondary antibodies, followed by DAPI for nuclear staining, and visualized under fluorescence microscopy (Dmi8, Leica, Wetzlar, Germany).

### 2.13. Statistical Analysis

All data were presented as mean ± standard deviation. All experiments were conducted at least three times independently. Student’s *t*-test was used for statistical differences between the two groups. The differences among three or more groups were analyzed using one-way and two-way ANOVA with Tukey’s multiple comparison test. For all comparisons, a *p*-value < 0.05 was considered statistically significant (* *p* < 0.05, ** *p* < 0.01, *** *p* < 0.001, **** *p* < 0.0001).

## 3. Results and Discussion

### 3.1. Preparation and Acetylation of BSP and GM

**Figure 1:**
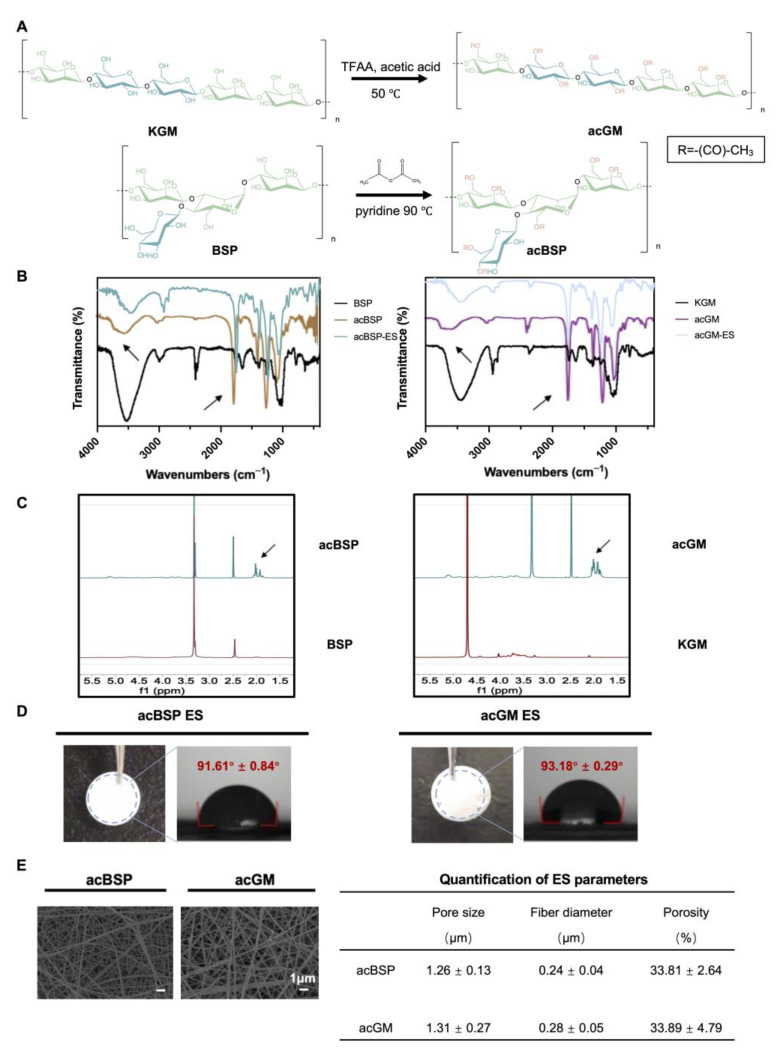
Preparation of acBSP and acGM as electrospun scaffolds. (**A**) schematic of the acetylation of KGM and BSP. (**B**) FT-IR spectra of BSP and KGM before and after acetylation (the arrow at 3500 cm^−1^ represents hydroxyl and the arrow at 1750 cm^−1^ represents carbonyl). (**C**) ^1^H-NMR spectra of BSP and KGM before and after acetylation (the arrow represents the methyl proton group of the acetyl group). (**D**) Gross view of electrospun scaffolds made of acBSP or acGM and measurement of contact angles. (**E**) SEM images of electrospun scaffolds made of acBSP or acGM and quantification of pore radius, fiber diameter and porosity. Data are presented as mean ± standard deviation (n = 3).

BSP (188.8 kDa) was extracted according to our in-house protocol, while KGM (201.7 kDa) was purchased from Shimizu Chemical Corporation. Both polysaccharides share a glucomannan backbone and only show differences in structural arrangement: they differ in linear or branched forms (Figure 1A). BSP has large branches, which are found in one of every 2–4 monosaccharide units, while KGM was used as the linear control in this study.

Subsequently, BSP and KGM were modified by acetylation, which on the one hand increases their hydrophobicity to facilitate the fabrication of the electrospun scaffolds. The hydrogen bonds are disrupted by the hydrophobic methyl groups, leading to higher viscosity, and the solubility was enhanced by the acetyl groups. Thus, their surface energy was reduced leading to better distribution in organic solvents which met one of the basic conditions for electrospinning [29]. On the other hand, introducing acetyl groups changed the spatial arrangement of polysaccharide chains, which may affect the biological activity of the polysaccharides. In addition, it has been proven that KGM with a specific degree of acetylation can exert the activity to induce macrophages into a pro-inflammatory state [30]. According to the FT-IR (Figure 1B), the signal of the hydroxyl peak at 3500 cm^−1^ was weakened while the peak at 1750 cm^−1^ was enhanced, which indicates that the hydroxyl group on the chain was replaced by a carbonyl group, confirming a successful acetylation. ^1^H-NMR further verified successful acetylation (Figure 1C), and the signal peak between 1.8 and 2.2 ppm can be attributed to the methyl proton group of the acetyl group. Next, the degree of acetylation of acBSP and acGM was shown to be comparable. The acBSP contains about 1.8 acetyl groups per sugar ring, while acGM has about 1.54.

### 3.2. Preparation and Characterization of acBSP and acGM as Electrospun Scaffolds

Having obtained acBSP and acGM with similar degrees of modification, electrospun scaffolds were fabricated from these two glycopolymers (Figure 1D). The contact angles of these materials were measured and data suggest that both samples had comparable hydrophobicity. Besides, the characteristic peaks in FT-IR results before and after the preparation of the scaffolds did not change, which indicated that the electrospinning process did not bring changes in the chemical properties of the material (Figure 1B). Furthermore, SEM results reveal that the electrospun scaffolds had similarities in surface morphologies, including fiber diameter, porosity, and pore radius (Figure 1E). Overall, the characterization studies suggest that despite the structural differences of the polysaccharide (linear and branched GM), both electrospun scaffolds had similar physicochemical properties.

### 3.3. Biocompatibility of Electrospun Scaffolds

To factor out the effects of residual organic solvents during scaffold preparation on cells and explore the biocompatibility of the scaffolds, the functional and behavioral effects on two cell lines were evaluated. Mouse macrophage cell line RAW264.7 and mouse embryonic fibroblast cell line NIH/3T3 were selected for these studies. The former is one of the most widely used in inflammatory cell models, and the latter is often used in fibrosis studies.

**Figure 2:**
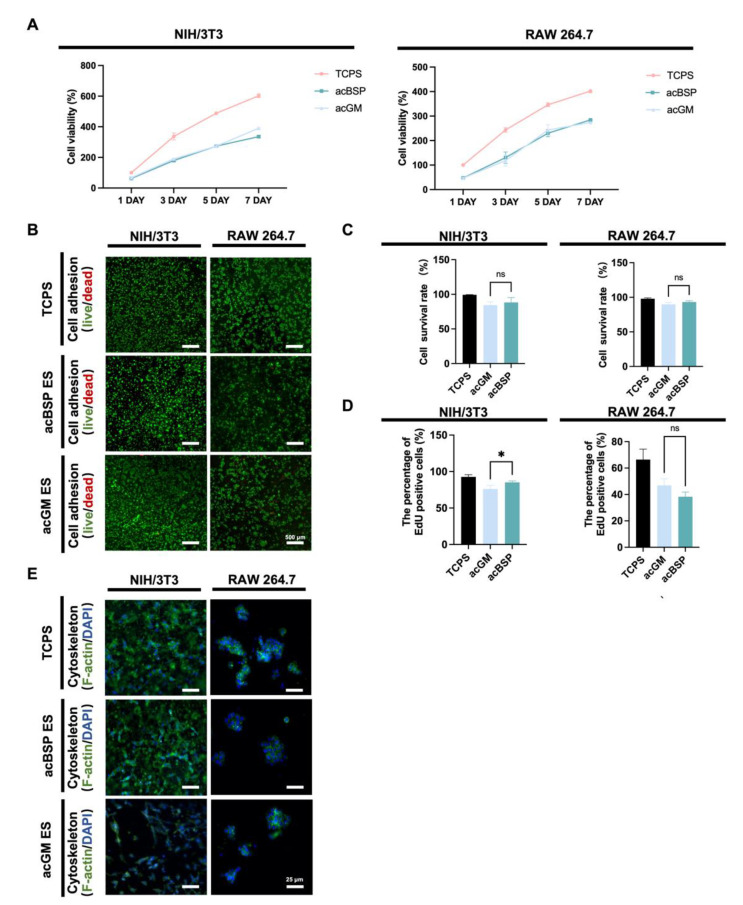
Biocompatibility of NIH/3T3 and RAW264.7 on electrospun scaffolds of acBSP and acGM. (**A**) Proliferation condition of NIH/3T3 and RAW264.7 on tissue culture polystyrene (TCPS) surface, acBSP scaffolds or acGM scaffolds. (**B**) Live/dead staining of cells cultured on different materials. Green: living cell. Red: dead cell; scale bar, 500 μm. (**C**) Quantification of the living cell ratio, by the average of living cell numbers as a percentage of total cells in three fields randomly selected from each group (ns: no statistically significant differences, n = 3). (**D**) Quantification of proliferation cells on TCPS surface, acBSP scaffolds or acGM scaffolds (* *p* < 0.05, n = 3). (**E**) Cytoskeleton staining of cells cultured on different materials. Green: F-actin. Blue: DAPI; scale bar, 25 μm.

According to the CCK8 results (Figure 2A), no significant differences were found between acBSP and acGM electrospun scaffolds on cell proliferation. This suggests that these scaffolds had no adverse effects on the growth of the cells of interest. Cellular adhesion was evaluated via fluorescence imaging studies. Having cultured the cells on different scaffolds for three days, the cells were stained with Calcein-AM/PI (Figure 2B) and quantitatively counted (Figure 2C). The results showed that both cells could achieve good adhesion on the surfaces of the two materials with high viability. Furthermore, a 12-h EdU proliferation test (Figure 2D) was performed on cells growing on the surfaces of either material. It was observed that NIH/3T3 achieved faster proliferation on the surface of acBSP, while RAW264.7 growth on acGM and acBSP had no statistical significance. The effects of culturing these cells on either material on the cell cytoskeleton were also studied (Figure 2E). RAW264.7 did not show an elongated or branched shape corresponding to the polarized state. Instead, it retained its classic spherical state. Moreover, NIH/3T3 also maintained its shape and achieved a good spread on the material surface. In summary, these in vitro experimental results demonstrate the good biocompatibility of the two materials for both types of cells.

### 3.4. Gene Expression of Macrophages and NIH/3T3 on Electrospun Scaffolds

**Figure 3:**
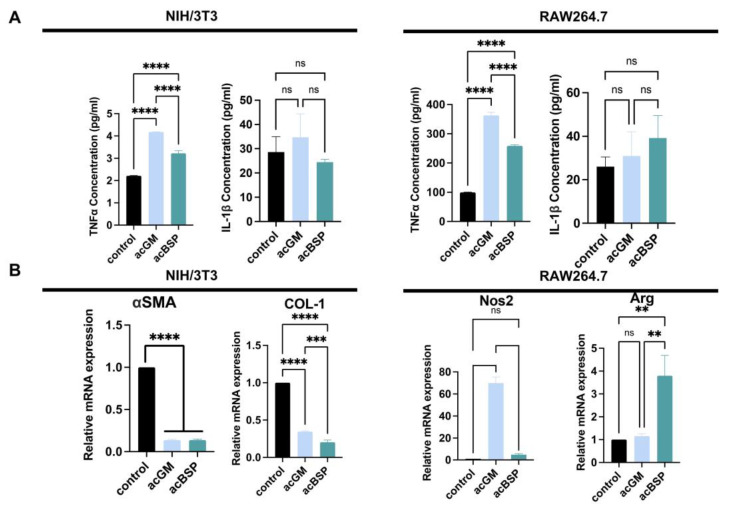
Gene expression analysis of NIH/3T3 and RAW264.7 on electrospun scaffolds of acBSP and acGM. (**A**) ELISA of supernatant of these cells seeded on TCPS and acBSP or acGM electrospun scaffolds (**** *p* < 0.0001, ns: no statistically significant differences, n = 3). (**B**) RT-qPCR analysis of the levels of representative genes in NIH/3T3 and RAW264.7 cells (** *p* < 0.01, *** *p* < 0.001, **** *p* < 0.0001, ns: no statistically significant differences, n = 3).

In order to explore the FBRs caused by these materials, the expression of FBR-related genes of RAW264.7 and NIH/3T3 on these materials was monitored. In addition, enzyme-linked immunosorbent assay (ELISA) was performed to determine the amounts of pro-inflammatory cytokine secreted by these different cells (Figure 3A). The results indicate that NIH/3T3 on acGM scaffolds produced a comparatively higher amount of tumor necrosis factor-α (TNF-α). However, due to the low secretion of TNF-α, there is no statistically significant difference. Similarly, the secretion of interleukin-1β (IL-1β) between acGM and acBSP electrospun scaffolds showed no statistically significant difference. As for RAW264.7 cells, the secretion of TNF-α on acGM was significantly higher than acBSP, indicating that acGM causes a greater inflammatory response. The secretion of IL-1β, however, showed no significant difference.

We picked up some representative genes and analyzed their changes individually by RT-qPCR. Firstly, a significant down-regulation of collagen I (COL-1) and alpha-smooth muscle actin (α-SMA) was verified in NIH-3T3 cultured on both acGM and acBSP (Figure 3B). These two markers are closely related to fibrosis, hence indicating that the scaffolds tested did not promote fibrosis in NIH-3T3 cells. We speculate that the soft physical properties of the electrospun scaffolds may change the mechanical microenvironment of cell growth, leading to changes in cell morphology. Besides, the significant up-regulation of nitric oxide synthase 2 (Nos2) verified in RAW264.7 cells cultured on acGM (Figure 3B) is consistent with our previous reports on acGM stimulation of macrophages to release pro-inflammatory cytokines [30]. Interestingly, although the pro-inflammatory cytokine had a four-fold up-regulation on the electrospun acBSP compared to the tissue culture polystyrene (TCPS) group, the corresponding anti-inflammatory cytokine arginine (Arg) also showed a four-fold up-regulation. The simultaneous up-regulation of both pro-inflammatory and anti-inflammatory cytokines reflects an inflammation-free environment.

### 3.5. Comparison of In Vivo Immune Responses of Electrospun Scaffolds

**Figure 4:**
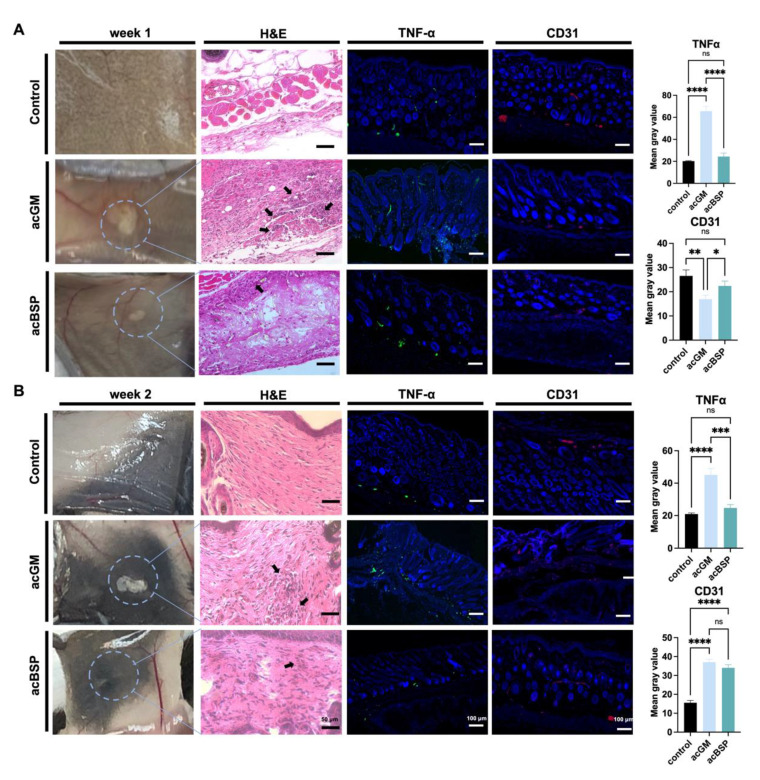
Evaluation of electrospun scaffolds of acBSP and acGM in vivo. (**A**) Assessment of the foreign body reaction of scaffolds on day 7 in vivo: haematoxylin and eosin staining (arrows represent inflammatory cell infiltration), TNFα staining and CD31 staining and quantification of them (* p < 0.05, ** p < 0.01, *** p < 0.001, **** p < 0.0001; ns: no statistically significant differences, n = 3). (**B**) Assessment of the foreign body reaction of scaffolds on the 14th day in vivo: haematoxylin and eosin staining (arrows represent inflammatory cell infiltration), TNFα staining and CD31 staining and quantification of them (* p < 0.05, ** p < 0.01, *** p < 0.001, **** p < 0.0001; ns: no statistically significant differences, n = 3).

Triggering an FBR is one of the simplest ways to investigate the immune response of a material. Thus, acBSP and acGM electrospun scaffolds were subcutaneously implanted on the back of mice to explore the FBR of the two materials in vivo. The mouse skin was excised for observation on days 7 and 14, respectively. Histological analysis of the removed tissue was performed to assess the different FBR elicited in vivo by the two materials. HE staining results on day 7 after implantation (Figure 4A) showed that inflammatory cells were infiltrated in the vicinity of the implanted materials and that more inflammatory cells were recruited in the acGM group than that of acBSP. Surprisingly, on day 14, an interesting phenomenon was observed (Figure 4B). In contrast to the acGM scaffold, which was still clearly observed, the electrospun acBSP scaffold was almost entirely absorbed by the body, and it was difficult to identify. The results of HE staining also showed that inflammatory cell infiltration close to the acGM scaffold remained, while the inflammation caused by acBSP electrospinning was gradually diminished. At the same time, in the healthy tissue of the control group, a typical arrangement of fibroblasts can be seen, with no inflammatory cells found.

The samples were then immunostained for TNFα to provide a clearer image of the FBR elicited. In the acBSP samples, the expression of TNFα was maintained at low levels on days 7 and 14, but the expression on day 7 was slightly higher than that on day 14. Its expression was also restricted to the area close to the sample-tissue interface throughout the 14 days. In the acGM samples, however, the expression of TNFα was very high on days 7 and 14. Despite a slight decrease of TNFα expression on day 14, the overall expression was significantly higher than acBSP samples, indicating that acGM elicits a stronger FBR in the body, leading to inflammation.

Besides, the expression of endothelial marker CD31 was significantly higher in the acBSP scaffolds on day 7, suggesting superior vascularization. Nevertheless, on day 14, the expression of CD31 was similar across both scaffolds, which may be attributed to the dampening of inflammation.

The diverse local immune responses may be caused by the discrete distribution of branches or monomers along a main chain of carbohydrates, thus leading to different FBR. Another point is that, compared to the branched polysaccharides, the linear chains of glycan moieties can be closely packed with one another more easily, forming hydrogen bonds between chains, leading to crystalline materials with a lower solubility in aqueous physiological environments. Besides, the acetyl functional modification of polysaccharides can be enzymatically digested in the local in vivo environments. Enzyme substrates are the primary targets of the enzyme’s active site, which is hydrolyzed to assist the degradation of polymeric materials in the body. In this way, different arrangements of sugar chains, i.e., different primary structures, may lead to acBSP becoming a more appropriate enzyme substrate, which makes it degrade faster in vivo [31]. At the same time, biomaterials that degrade faster will correspondingly cause inflammation to subside faster [32].

## 4. Conclusions

Our results suggest that implanted electrospun acGM leads to a stronger FBR than acBSP. As observed in the in vitro experiments, acGM could stimulate macrophages to release pro-inflammatory cytokines and cause more inflammation than acBSP. Our study suggests that naturally derived polysaccharides with a subtle structural difference may elicit a very different FBR. We also clarified the maturity and controllability of electrospinning technology as a polysaccharide processing technology. However, the mechanisms in which polysaccharides that possess identical sugar subunits but different structural arrangement trigger different immune responses remain to be elucidated. We speculate that such mechanisms may be related to different immune cell types and receptors. By understanding these underlying mechanisms, we can take advantage of these differences to achieve more fine-tuned regulation of cells by biomaterials and even innovate other types of polysaccharides in developing novel biomaterials.

## Data Availability

Not applicable.
